# Identification of most influential co-occurring gene suites for gastrointestinal cancer using biomedical literature mining and graph-based influence maximization

**DOI:** 10.1186/s12911-020-01227-6

**Published:** 2020-09-03

**Authors:** Charles C. N. Wang, Jennifer Jin, Jan-Gowth Chang, Masahiro Hayakawa, Atsushi Kitazawa, Jeffrey J. P. Tsai, Phillip C.-Y. Sheu

**Affiliations:** 1 Department of Bioinformatics and Medical Engineering, Asia University, Taichung, Taiwan; 2 Center for Artificial Intelligence in Precision Medicine, UAsia University, Taichung, Taiwan; 3 Department of EECS and BME, University of California, Irvine, USA; 4 Department of Laboratory Medicine, China Medical University Hospital, Taichung, Taiwan; 5 Center for Precision Medicine, China Medical University Hospital, Taichung, Taiwan; 6 Graduate Institute of Clinical Medical Science, School of Medicine, College of Medicine, China Medical University, Taichung, Taiwan; 7 NEC Solution Innovators, Koto-ku, Tokyo, Japan

**Keywords:** Gastrointestinal cancer, Text mining, Bi-LSTM-CNN-CRF, Influence maximization, Co-occurrence network

## Abstract

**Background:**

Gastrointestinal (GI) cancer including colorectal cancer, gastric cancer, pancreatic cancer, etc., are among the most frequent malignancies diagnosed annually and represent a major public health problem worldwide.

**Methods:**

This paper reports an aided curation pipeline to identify potential influential genes for gastrointestinal cancer. The curation pipeline integrates biomedical literature to identify named entities by Bi-LSTM-CNN-CRF methods. The entities and their associations can be used to construct a graph, and from which we can compute the sets of co-occurring genes that are the most influential based on an influence maximization algorithm.

**Results:**

The sets of co-occurring genes that are the most influential that we discover include RARA - CRBP1, CASP3 - BCL2, BCL2 - CASP3 – CRBP1, RARA - CASP3 – CRBP1, FOXJ1 - RASSF3 - ESR1, FOXJ1 - RASSF1A - ESR1, FOXJ1 - RASSF1A - TNFAIP8 - ESR1. With TCGA and functional and pathway enrichment analysis, we prove the proposed approach works well in the context of gastrointestinal cancer.

**Conclusions:**

Our pipeline that uses text mining to identify objects and relationships to construct a graph and uses graph-based influence maximization to discover the most influential co-occurring genes presents a viable direction to assist knowledge discovery for clinical applications.

## Introduction

Gastrointestinal (GI) cancer is the most common human tumors encountered worldwide [1]. These include colorectal cancer, gastric cancer, pancreatic cancer, and cancer of the liver and of the biliary tract. Although early-stage GI cancers are amenable to surgical resection with curative intent, the overall 5-year relapse rate remains high. The addition of neoadjuvant or adjuvant chemotherapy and radiation therapy only modestly improves the overall long-term survival [2]. Approximately 25% of GI cancers are diagnosed in an advanced stage, whereas another 25 to 50% of patients will develop metastases during the course of the disease [3]. GI cancers are still a leading cause of cancer death [4]. Therefore, it is imperative to explore potential effective influential genes to increase the number of patients qualified for curative treatments.

The increase in biomedical articles and the formation of various biomolecule interaction databases enable us to obtain diverse biological networks. These biological networks provide a wealth of raw materials for further understanding of biological systems, the discovery of complex diseases, and the search for therapeutic drugs [5]. Both text mining and network analysis have been applied to find the hidden biological knowledge and gene regulation rules behind the huge amount of information [6]. Biomedical text mining for extracting biomedical facts from biomedical literature has improved considerably [7]. It has four main phases:

1. Identify relevant literature that is known such as PubMed (https://pubmed.ncbi.nlm.nih.gov/);
2. Recognize biological entities that are mentioned in the literature (for example, genes and disease);
3. Enable specific facts that relate the entities to be pulled from the literature; and
4. Discover knowledge, where the extracted relationships are used to identify useful patterns from the literature.

On the other hand, network analysis has been used extensively in sociology to study the relationships and community structures in social data. Similarly, we can use network analysis to identify the key genes within a gene regulatory network [8].

In this study, we integrate deep learning and network analysis, including Bi-LSTM-CNN-CRF, influence maximization, and pattern-based approaches into a pipeline to automatically extract potential gene information from a collection of biomedical literatures and generate a ranked influential gene list composed of genes. Cho and Lee [9] propose herein an NER system for biomedical entities by incorporating n-grams with Bi-LSTM-CNN-CRF. The Bi-LSTM-CNN-CRF model achieves the best results, outperforming the previously decision tree and neural network. Therefore, we choice the Bi-LSTM-CNN-CRF model in our study. We use Bi-LSTM-CNN-CRF to identify and tag entities in the text as members of a set of predefined categories (such as diseases, chemicals, genes, etc.) and apply influence maximization to derive the sets of co-occurring genes with the highest influence on a set of cancer. We use the Molecular Signatures Database (MSigDB, https://www.gsea-msigdb.org/gsea/index.jsp) [10] to export the gene sets of interest to gene set files that can be used with the Gene ontology and pathway analysis. It is a collection of gene sets originally created for use with the Gene Set Enrichment Analysis (GSEA) software and DAVID (Database for Annotation, Visualization, and Integrated Discovery, https://david.ncifcrf.gov/tools.jsp) [11] to functionally enrich the revealed important biological associations across pathways, transcriptional control, gene ontologies, and other biological terms.

In the past, Zhu et al. [12] applied text mining to extract information from biomedical literatures to search and identify interactions between disease-associated biological units, conceive hypotheses from available data, and chart biological conduits. Jurca et al. [8] integrated text mining and social network analysis in order to identify new potential biomarkers for breast cancer. Chang et al. [13] reported a text mining-aided curation pipeline to identify potential biomarkers of cancer. Network analysis often uses influence maximization methods. In our application of influence maximization to genetic regulatory networks, the diffusion process represents a flow of information on the network, which opens up many applications in biomedical [14]. However most existing approaches, to our knowledge, do not address co-occurring gene suites as we do in this study.

## Material and methods

**Fig. 1:**
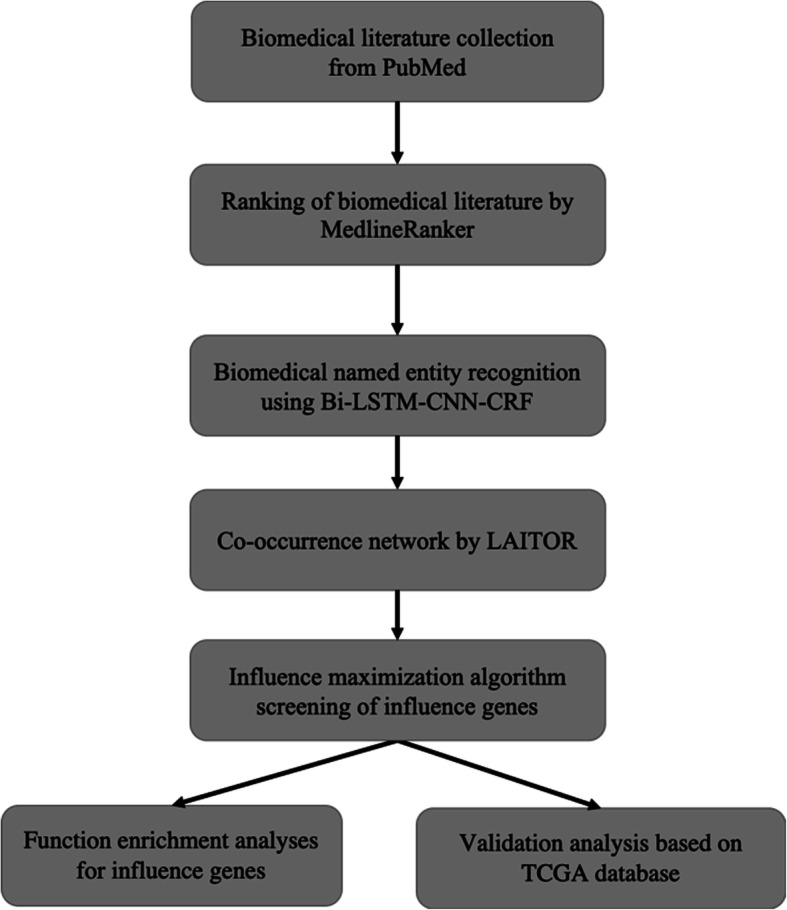
A schematic diagram of the developed curation pipeline

The workflow of the developed key gene extraction curation pipeline is shown in Fig. 1. We elaborate on each step in the following sub-sections.

The workflow of the developed loped curation pipeline is shown in Fig. 1. We use biomedical literature mining and graph-based influence maximization, and integrated bioinformatics database to identifying influential genes for GI cancers. First, we use “colon adenocarcinoma”, “liver hepatocellular carcinoma”, “pancreatic adenocarcinoma”, “stomach adenocarcinoma”, “stomach cancer”, “colorectal cancer”, “gallbladder cancer”, “liver cancer”, and “pancreatic cancer” as query keywords, we search the whole PubMed database. We then use their abstracts as the training set to train the literature ranking tool MedlineRanker. The trained MedlineRanker is used to rank the PubMed publications published in recent 10 years, and the top 1000 publications are selected to conduct the text mining procedures. Next, we adopt Named Entity Recognition (NER) and co-occurrence network for mining co-occurrences of gene pairs from the biomedical literature. To extract gene associated with the concept of GI cancers from the abstracts, we apply an influence maximization method to biological co-occurrence networks, aiming to discover each set of regulatory genes that together have the greatest influence on the network dynamics. Finally, we use MSigDB and DAVID to identify which gene suites reveal important biological associations across pathways and verify the expression levels of the influence genes among the GI cancer patients of the TCGA database.

### Datasets

#### Biomedical datasets

Gastrointestinal cancer refers to malignant conditions of the gastrointestinal (GI) tract and other organs involved in digestion, including the esophagus, stomach, biliary system, pancreas, small intestine, large intestine, rectum, and anus. In this study, we use data from four cancer types that belong to the Cancer Genome Atlas (TCGA) (https://portal.gdc.cancer.gov/) [15]. It provides the researcher with unprecedented amounts of molecular data along with clinical and histopathological information. We download level 1 clinical data and level 3 miRNA isoform sequencing raw counts. The corresponding data of TCGA-COAD, TCGA-LIHC, TCGA-PAAD, and TCGA-STAD are retrieved from TCGA. We use gene or isoform expression levels and survival days to analyze the GI cancer survival rate. The detailed lists of datasets, RNA-Seq, clinical and samples used are shown in Table 1. TCGA-COAD (Colon adenocarcinoma) are the third most commonly diagnosed cancers in both men and women and they account for 9 % of all cancer deaths. TCGA-LIHC (Liver hepatocellular carcinoma) is the most common form of liver cancer in the United States, making up more than 80% of cases. TCGA-PAAD (Pancreatic adenocarcinoma) is the most common form of pancreatic cancer, making up more than 80% of cases. TCGA-STAD (Stomach adenocarcinoma) is the 16th most common cancer in the United States, arises in the cells that line the stomach. All the data collected in TGCA are a part of a larger effort to build a research community focused on connecting cancer phenotypes to genotypes and clinical data.

**Table 1:** Cancer datasets used for our study

TCGA ID	Sample/Censored	Platform
TCGA-COAD (2)	461/460	RNA-Seq/ Clinical
TCGA-LIHC (4)	377/376	RNA-Seq/ Clinical
TCGA-PAAD (5)	185/177	RNA-Seq/ Clinical
TCGA-STAD (6)	443/443	RNA-Seq/ Clinical

#### Biomedical literature collection

We employ text mining to search for the genes related to four gastrointestinal cancers that are scattered in PubMed. Using the query “colon adenocarcinoma”, “liver hepatocellular carcinoma”, “pancreatic adenocarcinoma”, “stomach adenocarcinoma”, “stomach cancer”, “colorectal cancer”, “gallbladder cancer”, “liver cancer” and “pancreatic cancer”, we search the whole PubMed with titles including either of the four gastrointestinal cancer keywords. It should be noted that the literature search method employed may return articles that are less biological meaningful. Although our results demonstrate the usefulness of our approach, the accuracy of literature search may be improved by employing more sophisticated tools such as PubTator [16] and LitVar [17].

##### Stomach cancer

Search terms including “(“stomach neoplasms“[MeSH Terms] OR (“stomach“[All Fields] AND “neoplasms“[All Fields]) OR “stomach neoplasms“[All Fields] OR (“stomach”[All Fields] AND “cancer”[All Fields]) OR “stomach cancer”[All Fields])” are used in our search strategies. The publication date is limited to the last 10 years and a total of 35,097 articles are retrieved.

##### Pancreatic cancer

Search terms including “(“pancreatic neoplasms“[MeSH Terms] OR (“pancreatic“[All Fields] AND “neoplasms“[All Fields]) OR “pancreatic neoplasms“[All Fields] OR (“pancreatic”[All Fields] AND “cancer”[All Fields]) OR “pancreatic cancer”[All Fields])” are used in the search strategies. The publication date is limited to the last 10 years and a total of 42,397 articles are retrieved.

##### Liver cancer

Search terms including “(“liver neoplasms“[MeSH Terms] OR (“liver“[All Fields] AND “neoplasms“[All Fields]) OR “liver neoplasms“[All Fields] OR (“liver”[All Fields] AND “cancer”[All Fields]) OR “liver cancer”[All Fields])” are used in the search strategies. The publication date is limited to the last 10 years and a total of 99,061 articles are retrieved.

##### Colorectal cancer

Search terms including “(“colorectal neoplasms“[MeSH Terms] OR (“colorectal“[All Fields] AND “neoplasms“[All Fields]) OR “colorectal neoplasms“[All Fields] OR (“colorectal”[All Fields] AND “cancer”[All Fields]) OR “colorectal cancer”[All Fields])” are used in the search strategies. The publication date is limited to the last 10 years and a total of 95,800 articles are retrieved.

As the contents of these publications focus on GI cancer, we use their abstracts as the training set to train the literature ranking tool MedlineRanker [18], which ranks the biomedical literature according to the relevance of a topic learned from the training set. In this study, we use the training set (35,097 related GI cancer literature) and the test set (GI cancer publications published in the last 10 years from PubMed) to help identify the most relevant results that top 1000 publications *(P-value < 0.05)* related to GI cancer literature. Fontaine et al. [18] claim 1000 abstracts are appropriate for most of the topics, but providing more abstracts is likely to improve the precision. Of course, the more homogeneous are the abstracts related to a topic, the better the ranking will be.

### Biomedical named entity recognition

With the progress in deep learning, use natural language processing (NLP) extracting valuable information from biomedical literature has gained popularity among researchers, and deep learning has boosted the development of effective biomedical text mining models [19]. Biomedical named entity recognition is one of the most fundamental facilitators in biomedical text mining that aims to automatically classify biomedical entities, such as genes, proteins, chemicals and disease [20]. The gene recognition task is formulated as a sequential labelling problem, and a set of linear-chain CRFs are used to compute the probability associated with the corresponding hidden labelled sequence of a sentence [13].

In this study, we scan each entire article and partially match the text with the specific terms listed in a species dictionary, and then use full name-abbreviation information to extract the designated species symbol prefixed in a gene name. We use a recurrent neural network with bidirectional Long Short-Term Memory (biLSTM), Convolutional Neural Networks (CNN), and Conditional Random Fields (CRF) called Bi-LSTM-CNN-CRF. We first use convolutional neural networks (CNNs) to encode character-level information of a word into its character-level representation. CNN is an effective approach to extract morphological information from characters of words and encode it into neural representations. Second, we combine character- and word-level representations. Third, we feed them into biLSTM to model context information of each word. The biLSTM presents each sequence forwards and backwards to two separate hidden states to capture past and future information. Finally, we use a sequential CRF to jointly decode labels for the whole sentence.

The CRF is beneficial to consider the correlations between labels in neighborhoods and jointly decode the best chain of labels for a given input sentence. The biLSTM and CNN have increasingly been employed for biomedical Named Entity Recognition (NER), yielding state-of-the-art performance at the time of their publications. Within these models, biLSTM and CNN are used to learn the optimal contextual vector representations of every linguistic unit in a sentence. They are taken as the input to a state-of-the-art advanced sequence labeling model called the CRF Linguistic units that are initialized with low dimensional continuous vector representations that are pretrained from extremely huge amount of unlabeled text. Some models also take as input character-level embeddings of words to their biLSTM models, bringing further outperformance to their biomedical NER models. Dang et al. [21] propose Bi-LSTM-CNN-CRF on the BioCreative V Chemical Disease Relation (BC5 CDR) corpus. Bi-LSTM-CNN-CRF achieves F1 of 93.14 and 84.68% for chemical and disease named entity recognition, respectively; while using the NCBI disease corpus, its F1 for the disease named entity recognition is 84.41% and the gene named entity recognition on FUS-PRGE is 87.62%.

We use word embedding of word representation in this study which is trained from a large amount of text [22]. We use PyTorch library to implement Bi-LSTM-CNN-CRF. To consider mitigating the overfitting problem, in this study we apply dropout on the weight vectors directly to mask the final embedding layer before the combinational embedding feed into the bi-directional LSTM. We fix the dropout rate at 0.25. The learning rate is set to 0.001. The CNN hidden layer size is 100. The decay rate is set to 0.05. The batch size is set to 10 and the epoch number is set of 100 to achieve good performance on our model. We also use the early stopping strategy with patience 15 to avoid overfitting the early stopping monitored weighted F1-scores on validation sets.

An important limitation of biomedical named entity recognition has been the lack of results from full text literature [23, 24], and studies have shown that biomedical named entity recognition efforts that are limited to abstracts may miss the important knowledge present in the full text [17, 25]. Unfortunately, full text articles are more complex than abstracts and they are 40 times longer, making them more difficult for biomedical named entity recognition. Moreover, in the past full text has been significantly less available than abstracts. However, the availability of full text articles for biomedical named entity recognition has recently increased, with the percentage of articles in PubMed Central available for biomedical named entity recognition approaching 80% [26].

### Co-occurrence network

In this study, we apply text mining to articles available in PubMed to generate a list of gene/protein co-occurrences related to gastrointestinal cancer interactions. We use LAITOR [27] as the text mining engine to extract sentences with co-occurring genes. The method accounts for the position of the co-occurring terms within sentences or abstracts. According to the semantic structure of each sentence and the whole abstract, the genes co-occurring with the customized concepts are likely gastrointestinal cancer reported in the biomedical literature. First, LAITOR uses the NLPort program as the information extraction tool [28] to tag the biomedical literature for biomedical entities using a species-specific dictionary composed of symbols and synonyms for the genes which include non-redundant names and alternative names from the corresponding UniPortKB recodes [29]. Next, LAITOR identifies gene interaction terms in the test of the biomedical literature according to a dictionary of gene interaction terms. Finally, co-occurrences between the previously identified biomedical entities are categorized into four types [30]:

1. Two entities co-occur in an abstract (type 4)
2. Two entities co-occur in a sentence (type 3)
3. Two entities co-occur in a sentence with an interaction term (e.g., activates, induces, inhibits) anywhere in the sentence (type 2)
4. Two entities co-occur in a sentence with an interaction term in between the entity names (type 1).

In this study, we do not handle negations, e.g., a sentence such as “gene A does not bind gene B”. One possibility is to try to recognize these sentences in order to just avoid them as it is difficult to identify if the negation refers to the information being extracted. Also we do not consider co-occurrence across documents and only consider the number of times two words co-occur in the same sentence. The genes pairs are categorized into four types of relevance degrees. Based on the degree of relevance to the customized concepts, we set their confidence scores to 0.25,0.50, 0.75, and 1 for types 4, 3, 2, and 1, respectively.

The adjacency matrix *a*_*ij*_ corresponding to the connection strength between each pair of nodes is calculated as follows:

$$ {s}_{ij}= correlation\ \left({x}_i,{x}_j\right)\mid {a}_{ij}={S}_{ij}^{\beta } $$

where *X*_*i*_ and *X*_*j*_ are vectors of expression values for genes *i* and *j*, *s*_*ij*_ represents the Pearson correlation coefficient of gene *i* and gene *j*, and *a*_*ij*_ encodes the network connection strength between gene *i* and gene *j.* We consider the genes for types 4, 3, 2 and 1 as gastrointestinal cancer genes and set their confidence scores ≥0.90 and target species on *Homo sapiens*.

### The influence maximization problem

In this study, we apply an influence maximization method to biological co-occurrence networks, aiming to discover each set of regulatory genes that together have the greatest influence on the network dynamics.

The traditional models include Linear Threshold Model (LTM) [31] and Independent Cascade Model (ICM) [32]. These existing influence models are binary (i.e., has influence or not). We propose a new model based on the probability of influence. It is consequently not discrete. Another problem with the traditional approaches is that they do not have any way of representing different classes of nodes or edges. We propose using colors on nodes and/or edges to represent different classes, and we study the IM problem in the context of “colored” graphs [29].

Consider a undirected graph *G* where each edge (*a,b*) carries a weight in the range (0, 1) that designates the probability of influence from node *a* to node *b*. Note that the weight of an edge should not be 0 because if there is no influence between the two end nodes, that edge should not exist. The Strongest Influence Path (SIP) problem finds the path from a given source node *s* to a given destination node *d* that has the strongest probability. The probability of influence for a path from *s* to *d* is calculated by multiplying the influence probabilities of all the edges on the path. The Influence Maximization (IM) problem finds s nodes which can influence G maximally.

The following heuristic algorithm is for the IM-SIP problem which is NP-complete [33]. The heuristic algorithm is greedy. It builds the set of seed nodes incrementally – in every iteration it brings in a new node that, combined with the nodes in the current set of seed nodes, has the highest combined influence on the remaining nodes.

1. Step 1: Compute the influence of each node on the entire graph.
2. Step 2: Sort the nodes based on their influence value.
3. Step 3: From the sorted list, choose the node from the top (node with the highest influence) to be a seed node.
4. Step 4: Among the nodes that are not in the set of seed nodes, for each one of them compute the combined influence of the set of seed nodes on the other nodes. Sort these nodes based on the influence value.
5. Step 5: With the node with the highest combined influence value with the existing seed nodes, add it to the set of seed nodes.
6. Step 6: Repeat steps 4 and 5 until all *s* seeds are selected.
7. Step 7: Return *s* seed nodes selected from the list.

The correctness and error bounds of the heuristic are proved in [33]. In the past, some studies apply network analysis methods to gene regulatory networks to solve the influence maximization problem or develop gene regulatory networks. Hashimoto et al. [34] develop an algorithm to grow small genetic-regulatory subnetworks from a smaller number of genes of interest, or a seed set of genes. Their algorithm is based on the strength of the connection between prospective genes to be added and the subnetwork in the current stage of the algorithm. Hecker et al. [35] propose an algorithm for starting with a seed network of genes and expending it to query the composite correlational network in a way that allows users to rank other genes for possible inclusion in an extended seed network. Gibbs and Shmulevich [36] apply IM methods to biological networks to discover the set of regulatory genes with the greatest influence on network dynamics. An influence ranking on genes is produced by solving the IM problem over different numbers of source nodes and is compared to other metrics of network centrality. Nalluri et al. [37] apply information diffusion theory to quantify the influence diffusion in a miRNA (MicroRNA)-miRNA regulation network across many disease classes. Their method regulates the specific miRNAs critical diseases which perform an underlying part in their signing cascade and therefore may regulate disease progression. Although some use IM methods on gene regulatory networks, no work has the flexibility to customize it to specific set of genes or diseases. For example, our approach supports queries such as “Find 2 disease that are the most closely related to a gene set [P53, CD44, CDC6, STAT3].” and “Find 5 genes that are the most closely related to a disease set [“colorectal cancer”, “liver cancer”]” that could not be solved using existing approaches.

In applying the above algorithm, we use a graph of 487 nodes (genes) and 1626 edges (co-occurrences) and set the edge weights to 1 (type 1), 0.75 (type 2), 0.5 (type 3), and 0.25 (type 4), respectively.

## Results

### Validation of the co-occurrence network

To validate the efficiency of our co-occurring method in recognizing individual gene-gene interactions, we use a gene-gene interaction database extracted from the AIMed corpus ([38], ftp://ftp.cs.utexas.edu/pub/mooney/bio-data/). This dataset contains 307 gene-gene interactions manually extracted from 174 PubMed abstracts. These abstracts are analyzed by our co-occurring method. They are pooled with a recall of 89% (201/255) and a precision of 91% (201/222) of AIMed, and our co-occurring method is applied to this pooled set of 225 true positive gene-gene interactions. The results show that our co-occurring method has a recall of 91% (205/225) and a precision of 90% (180/200). In this study, we analyze a thematic selection of GI cancer articles consisting of 35,097 literatures related to GI cancer in the context of gene processing and aggregation surrounding gene-gene interactions. By removing those gene pairs with a relevance rate less than or equal to 5, a total of 487 gene terms and 1626 interactions are identified from the 35,097 literatures.

### Identification of influential genes

**Fig. 2:**
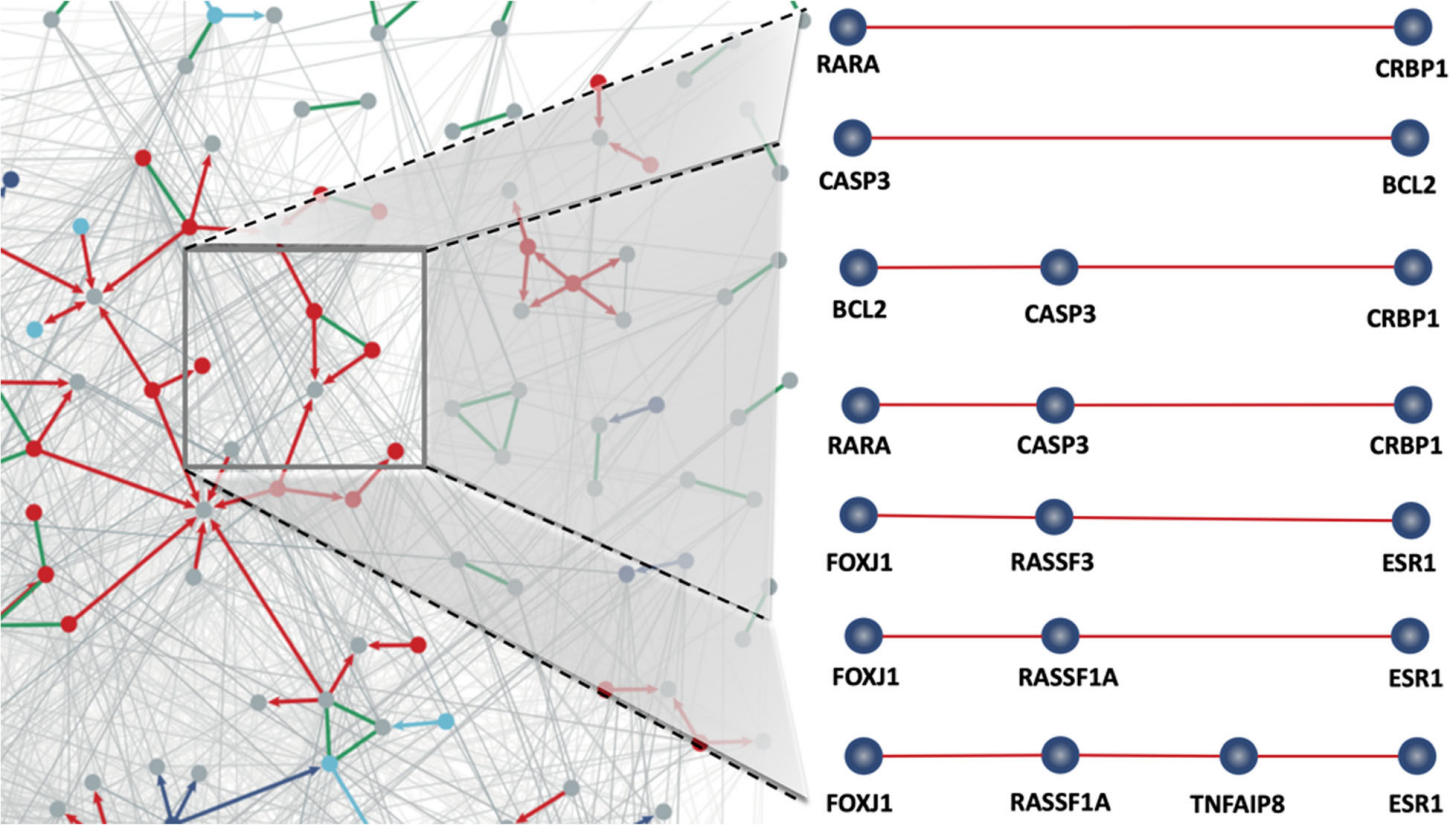
A GI cancer network derived from abstracts that are stored in PubMed, using co-occurrence and text mining

In this study, a total of 487 genes related to gastrointestinal cancer are extracted from 272,355 PubMed articles. With the influence maximization model discussed, an influence ranking on genes is produced. In our experiments, we set the number of target seed nodes (genes) from 2 to 5 for networks of 2 to 4 diseases. Among all the possible (gene-suite, disease-suite) pairs, those whose relevance are significant (7) are included in the result. For example, the gene suite (11,300,473) and disease suite (5,6) have a relevance of 2.625 which is considered significant among others. We remove those gene pairs with a relevance rate less than or equal to 5 and find seven sets of regulatory influence genes with the greatest influence on the GI cancer network (Fig. 2).

### Influential genes from functional and pathway enrichment analysis

We first conduct literature review to validate their importance and potential as clinical genes:

#### retinoic acid receptor alpha

The RARA gene represents a nuclear retinoic acid receptor. It has been implicated in the regulation of development, differentiation, apoptosis, and transcription of clock genes. In a recent study, Xiang et al. [39] find that RARA is a drug sensitive biomarker of ERBB2-targeted treatment. ERBB2-related pathways can help us finding sensitive molecules and potential combined therapeutic targets of ERBB2-targeted therapy for gastric cancer.

#### Cellular retinol-binding protein 1

The CRBP1 gene is encoded in the carrier protein involved in the transport of retinol (vitamin A alcohol) from the liver storage site to the peripheral tissue. In a previous study, colorectal and gastric adenomas frequently display methylation of the CRBP1 promoter region. The percentage found in the invasive colorectal and gastric tumors suggests that methylation-associated inactivation of CRBP1 is an early event in human tumorigenesis. Also aberrant methylation of CRBP1 has predictive value [40].

#### Caspase 3

CASP3 is a cysteine-aspartic acid protease that plays a central role in the execution-phase of cell apoptosis. Qiang et al. [41] discover Caspase-3 protein levels are upregulated in colorectal cancer tissues. Furthermore, high expressions of Caspase-3 are correlated with decreased overall survival and unfavorable clinicopathologic characteristics. Cox regression analysis shows that high Caspase-8 and Caspase-3 expressions are independent negative markers of overall survival. The result suggests that Caspase-3 expressions in tumor tissues are novel candidate prognostic markers for colorectal cancer patients.

#### BCL2, apoptosis regulator

BCL2 is a key regulator of apoptosis whose dysregulation can cause various pathological consequences including the development of cancer [42]. In a meta-analysis study, BCL2 high expression is significantly correlated with favorable overall survival, better disease-free survival, and recurrent free survival. Hence, BCL2 may be a valuable prognostic-predictive marker in colorectal cancer [43].

#### Forkhead box J1

FOXJ1 is a forkhead transcription factor that has been previously studied mostly as a ciliary transcription factor. In a recent report, an increased expression of FOXJ1 associated with the clinical stage, metastasis of lymph node, and invasion depth in colon cancer suggest FOXJ1 is a tumor promoter in colorectal cancer. The results suggest that increased FOXJ1 contributes to the progression of colorectal cancer, which might be associated with the promotion effect of β-catenin nuclear translocation, and it may be a novel therapeutic target in colorectal cancer [44].

#### Ras association domain family member 3/ Ras association domain family member 1

RASSF is a family of 10 members (RASSF1–10) implicated in a variety of key biological processes, including cell cycle regulation, apoptosis, and microtubule stability. The RASSFs have been implicated in colorectal cancer and several family members are now thought to be tumor suppressors. In particular, RASSF1A, RASSF3, and methylation have been associated with colorectal cancer development, although the mechanisms of action remain poorly understood. RASSF1 and RASSF3 have been considered as potential biomarkers and for the development of new targeted therapies for colorectal cancer [45].

#### Estrogen receptor 1

Caiazza et al. [46] analyze the estrogen pathway as a possible therapeutic avenue in colorectal cancer. The experimental evidence explains the cellular and molecular mechanisms of estrogen-mediated protection against colorectal tumorigenesis and suggests that ESR1 future challenges and potential avenues for colorectal cancer targeted therapy.

#### TNF alpha induced protein 8

In a recent study, TNFAIP8 has been associated with the tumorigenicity of gastric cancer. The decreased expression of TNFAIP8 inhibits the growth, invasion and migration of gastric cancer. It is a meaningful approach for treating human gastric cancer. In addition, the expression levels of TNFAIP8 may be considered as a biomarker of gastric cancer [47].

We use MSigDB and DAVID to identify which gene suites reveal important biological associations across pathways, transcriptional control, gene ontologies, and other biological terms associated with the set of co-occurring genes. All genes are uploaded to gene ontology (GO) and Kyoto encyclopedia of gene and genome (KEGG) pathway enrichment analyses, with a cutoff of *p*-value < 0.05 established for significant biological process and pathway, respectively. The influence genes can be categorized into three functional groups: Biological Process (BP), Cellular Component (CC), and Molecular Function (MF). The influence genes in the BP group are mainly enriched in signal transduction, apoptosis, and regulation of nucleobase; the influence genes in the MF group are mainly enriched in transcription factor activities; the influence genes in the CC group are significantly enriched in cytoplasm, nucleus, and mitochondrion. According to Kyoto Encyclopedia of Genes and Genomes (KEGG) pathway analysis, our results demonstrate that these genes are mainly involved in PAR1-mediated thrombin signaling events, VEGF and VEGFR signaling networks, Retinoic acid receptors-mediated signaling, BMP receptor signaling, Apoptosis, p53 pathway, ATR signaling pathway, and the regulation of RAC1 activities. The transcriptional analysis results suggest that these genes are highly associated with transcription factors such as SP1, SP4, IRF1, RREB1, EGR1, HENMT1, and NHLH1. Some of these gene terms, pathways, and transcription factors are well known to be associated with GI cancers. In addition, at least 487 of the seven sets of regulatory influence genes are associated with the biomedical literature on one or more cancer types (Table 2).

**Table 2:**
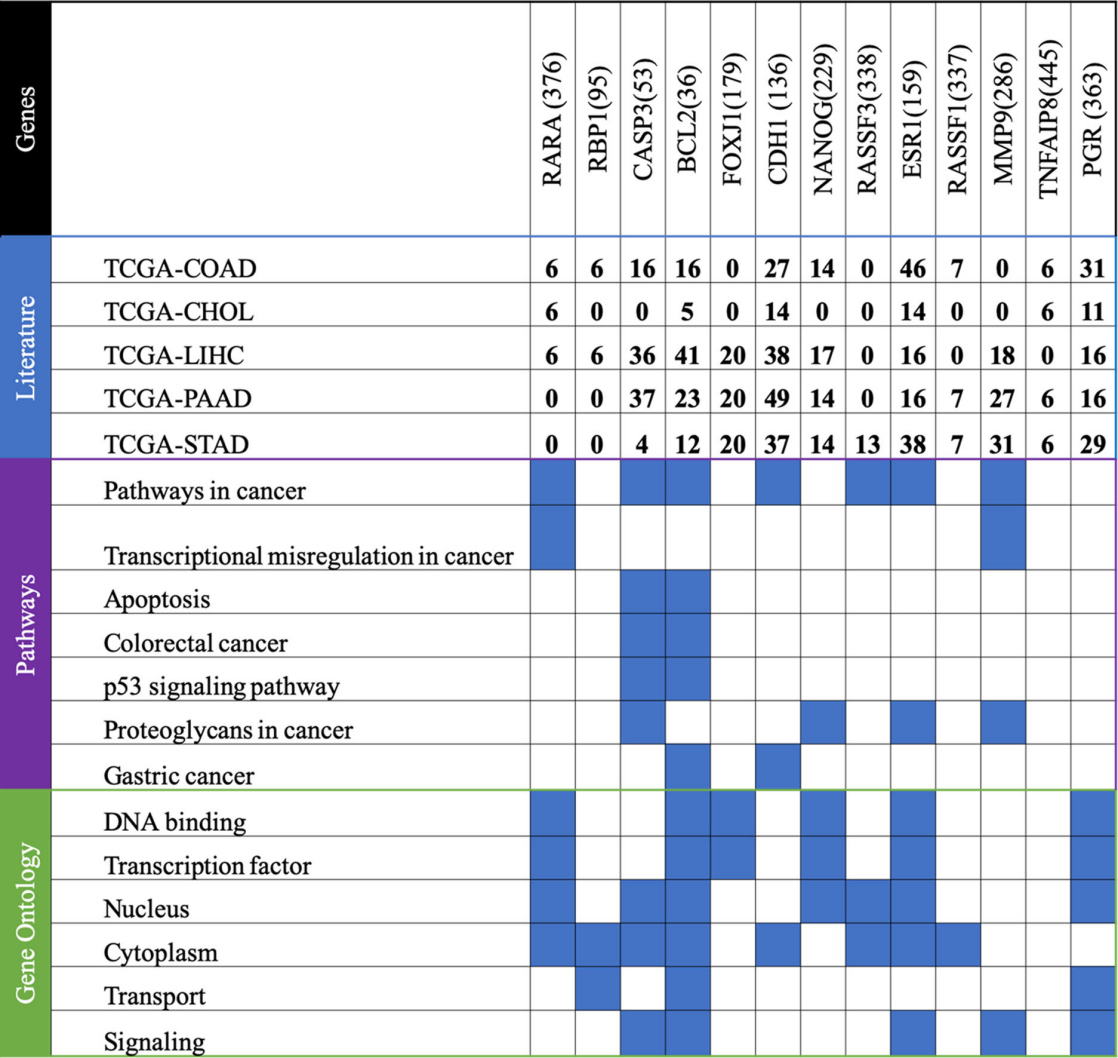
Associations of genes with GI cancer based on the literature, gene ontology, pathway, and transcription factor enrichment analysis

### Identification and validation of influential genes

To determine the significance of the concordance index values, we generate a null distribution composed of 1517 random models of 50 genes for the TCGA datasets used. To assess the concordance index prediction of the influential genes in datasets other than TCGA, we use SurvExpress [48] which provides evaluations of gene lists across cancer types. For this study, we use normalized datasets that include overall survival times and only those studies containing more than 26 samples. We also compare the concordance index values of the co-occurring influence gene suites discovered by us with those of other multi-cancer sets of influence genes reported in the biomedical literature.

**Fig. 3:**
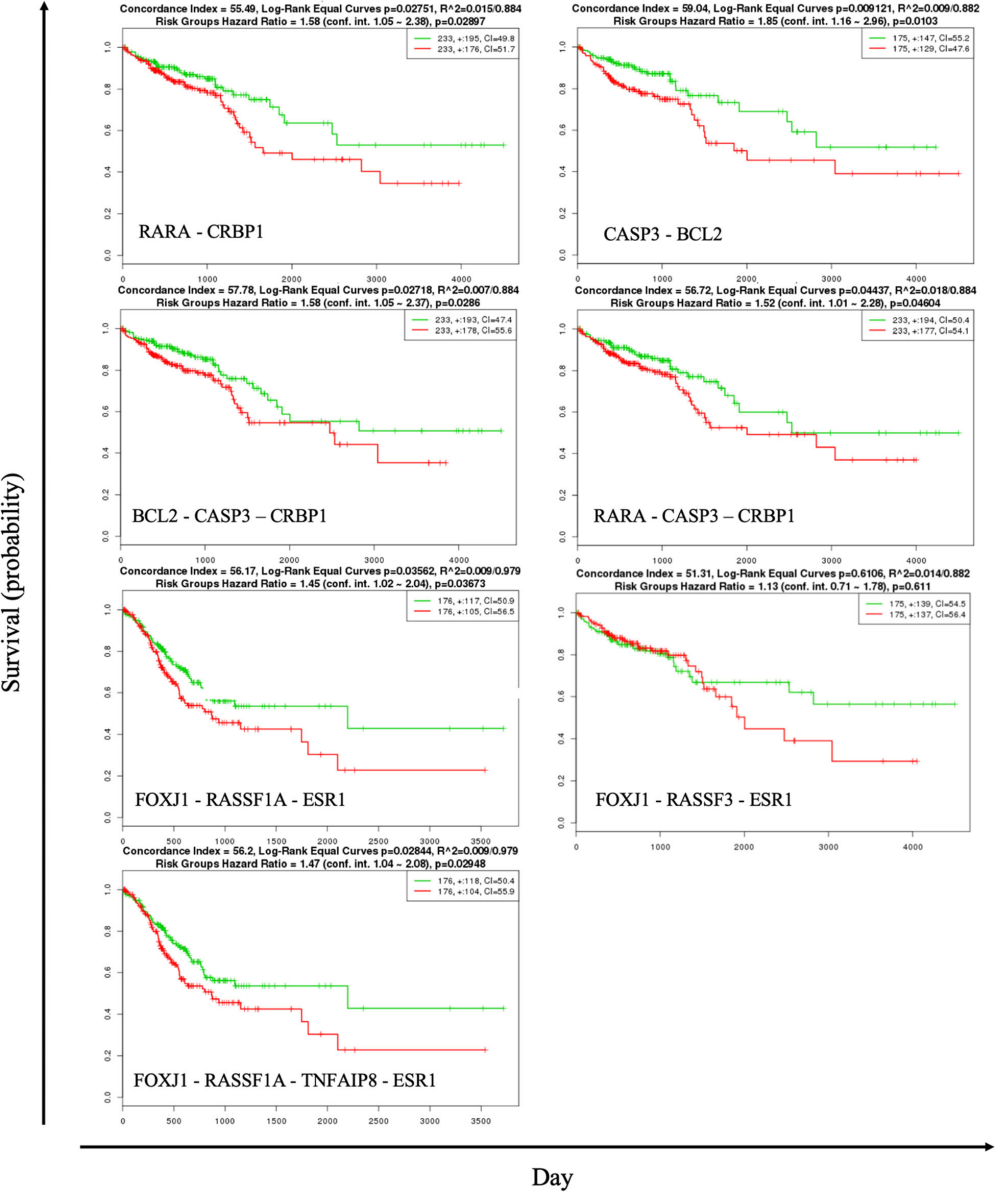
Kaplan-Meier survival curves of censored Cox analysis for TCGA colon adenocarcinoma, TCGA liver hepatocellular carcinoma, TCGA pancreatic adenocarcinoma and TCGA stomach adenocarcinoma by maximized influencing genes risk group (SurvExpress). Red lines represent the samples with a highly expressed gene and the green line represents samples with a lowly expressed gene

The identified seven sets of regulatory influence genes with the highest influence on the GI cancer network include RARA - CRBP1, CASP3 - BCL2, BCL2 - CASP3 – CRBP1, RARA - CASP3 – CRBP1, FOXJ1 - RASSF3 - ESR1, FOXJ1 - RASSF1A - ESR1, FOXJ1 - RASSF1A - TNFAIP8 - ESR1 with 9 constituent genes RARA, CRBP1, CASP3, BCL2, FOXJ1, RASSF3, ESR1, RASSF1A and TNFAIP8. These influence gene suites are able to discriminate low- and high-risk groups efficiently in the GI cancer through statistical association, i.e., those cases with a mutation in each of the 7 suites statistically have a lower survival rate compared to those cases without a mutation. The results show that the influence genes are able to separate risk groups characterized (see the Kaplan-Meier and box plots respectively in Fig. 3). Patients with high RARA - CRBP1, CASP3 - BCL2, FOXJ1 - RASSF3 - ESR1, FOXJ1 - RASSF1A - ESR1, FOXJ1 - RASSF1A - TNFAIP8 - ESR1 expressions are significantly associated with better overall survival compared to those with low expressions. The BCL2-CASP3-CRBP1 and RARA-CASP3-CRBP are the log-rank test of the difference in risk groups that is not significant. A limitation that mathematically it is possible that our approach may fail to discover some other gene suites that were reported to be influential. We are not able to identify such misses so far to our knowledge.

## Conclusions

With the huge amount of available data from clinical studies on GI cancer, a proper data curation and pipelined platform is required to help researchers discover potential influence genes from existing biomedical literatures. In this study, we introduce a curation pipeline developed for mining influence genes of GI cancers. The developed curation pipeline employs Bi-LSTM-CNN-CRF and co-occurrence network components to extract GI cancer genes from a large collection of biomedical literature and use a graph-based influence maximization algorithm to develop a panel of influence genes for GI cancer monitoring. Seven sets of regulatory influence genes including RARA - CRBP1, CASP3 - BCL2, BCL2 - CASP3 – CRBP1, RARA - CASP3 – CRBP1, FOXJ1 - RASSF3 - ESR1, FOXJ1 - RASSF1A - ESR1, FOXJ1 - RASSF1A - TNFAIP8 - ESR1 are identified and validated in association with the progression of GI cancer. Mathematically it is possible that our approach may fail to discover some other gene suites that were reported to be influential. We are not able to identify such misses so far to our knowledge.

We use CNN with different filter widths to extract character features and a word-level BiLSTM for sequence modelling which takes both word embeddings and character features as inputs. We pre-train the weights of the biomedical named entity recognition model using a bidirectional language model such that the architectures of both models are the same except the top decoder layer. In influence maximization, the set of influential genes seem to have a topologically advantageous position. We can speculate that they might be useful selections that impact the influential nodes, thereby affecting normal information flow and having a strong effect on the gene network, potentially leading to cancer states. Discovering the minimum sets of biomedical entities that have the greatest influence in a gene network could lead to further understanding of how network dynamics is associated with GI cancer. An interesting goal in the future would be to extract the evidence identified through the pipeline. On the whole, we believe that our curation pipeline can help biomedical researchers by reducing the time and effort spent on article collection and data analysis.

## Data Availability

All available data were analyzed in this study. They can be found at TCGA (https://portal.gdc.cancer.gov/) and PubMed (https://pubmed.ncbi.nlm.nih.gov/).
